# Evaluation of Immunodiagnostic Tests for Leprosy in Brazil, China and Ethiopia

**DOI:** 10.1038/s41598-018-36323-1

**Published:** 2018-12-18

**Authors:** Anouk van Hooij, Elisa M Tjon Kon Fat, Moises Batista da Silva, Raquel Carvalho Bouth, Ana Caroline Cunha Messias, Angélica Rita Gobbo, Tsehaynesh Lema, Kidist Bobosha, Jinlan Li, Xiaoman Weng, Claudio G. Salgado, John S. Spencer, Paul L. A. M. Corstjens, Annemieke Geluk

**Affiliations:** 1 Department of Infectious Diseases, Leiden University Medical Center, Leiden, The Netherlands; 2 Department Cell and Chemical Biology, Leiden University Medical Center, Leiden, The Netherlands; 3 Laboratório de Dermato-Imunologia, Instituto de Ciências Biológicas, Universidade Federal do Pará, Marituba, Pará Brazil; 4 Armauer Hansen Research Institute, Addis Ababa, Ethiopia; 5 Guizhou Provincial Center for Disease Control and Prevention, Guiyang, Guizhou China; 6 Beijing Tropical Medicine Research Institute, Beijing, China; 7 Department of Microbiology, Immunology and Pathology, Colorado State University, Fort Collins, USA

## Abstract

Leprosy remains persistently endemic in several low- or middle income countries. Transmission is still ongoing as indicated by the unabated rate of leprosy new case detection, illustrating the insufficiency of current prevention methods. Therefore, low-complexity tools suitable for large scale screening efforts to specifically detect *M. leprae* infection and diagnose disease are required. Previously, we showed that combined detection of cellular and humoral markers, using field-friendly lateral flow assays (LFAs), increased diagnostic potential for detecting leprosy in Bangladesh compared to antibody serology alone. In the current study we assessed the diagnostic performance of similar LFAs in three other geographical settings in Asia, Africa and South-America with different leprosy endemicity. Levels of anti-PGL-I IgM antibody (humoral immunity), IP-10, CCL4 and CRP (cellular immunity) were measured in blood collected from leprosy patients, household contacts and healthy controls from each area. Combined detection of these biomarkers significantly improved the diagnostic potential, particularly for paucibacillary leprosy in all three regions, in line with data obtained in Bangladesh. These data hold promise for the use of low-complexity, multibiomarker LFAs as universal tools for more accurate detection of *M. leprae* infection and different phenotypes of clinical leprosy.

## Introduction

Leprosy is a debilitating, infectious disease caused by *Mycobacterium leprae* (*M. leprae*) causing skin and nerve damage often leading to lifelong handicaps. The continued transmission of *M. leprae* accounts for approximately 200,000 new cases each year. Pockets of high endemicity where intense transmission is witnessed are still present^1^. Leprosy diagnosis mainly relies on detection of clinical symptoms^2^, which can take up to 20 years to manifest^1^. Moreover, the majority of infected individuals will never progress to disease but instead develop adequate immunity to eventually clear *M. leprae* or remain asymptomatically infected^3^. However, individuals from the latter group may still be accountable for transmission of *M. leprae* bacteria, particularly to close contacts. To reach worldwide elimination of leprosy abrogation of transmission of *M. leprae* is a top priority for leprosy research. Approaches that support detection of *M. leprae* infected individuals without clinical symptoms are therefore vital to achieve that goal.

Due to this inter-individual variability in immunity against *M. leprae*, diagnostic tests merely detecting antibodies against *M. leprae* specific antigens such as phenolic glycolipid I (PGL-I) or the LID-1 protein^4–8^ are not adequate as stand-alone tests to detect (early) disease since antibody detection tests identify mainly multibacillary (MB) leprosy patients with high bacillary loads (BI: bacillary index) which only cover part of the leprosy disease spectrum. On the other part of the spectrum, paucibacillary (PB) leprosy displays a dominant cellular phenotype showing restricted anti-*M. leprae* antibody production^9^. The MB/PB classification endorsed by WHO is based on the number of skin lesions and nerve involvement^1^. Alternatively, the Ridley-Jopling classification system^10^ identifies five disease types: tuberculoid (TT), borderline tuberculoid (BT), borderline (BB), borderline lepromatous (BL) and lepromatous leprosy (LL).

In 2016, 59% of the new cases worldwide were diagnosed with MB leprosy with ratios of MB and PB patients varying per endemic region^11^. Since PB cases are generally not detected using serological tools for anti *M. leprae* antibody detection, additional biomarkers are needed to identify the remaining 41% of PB leprosy patients. Moreover, as not all PGL-I seropositive individuals will develop disease, new diagnostic tests should be based on disease- and infection specific biomarkers allowing the distinction between individuals requiring therapeutic or prophylactic therapy, respectively. Tests based on signatures combining humoral- and cellular biomarkers may help to guide administration of postexposure prophylaxis (PEP), a currently introduced strategy aimed at reduction of transmission by *M. leprae* infected individuals without clinical symptoms of leprosy^12,13^.

In Bangladesh we previously demonstrated, using a field-friendly lateral flow assay (LFA)^7,14,15^, that combined detection of a humoral immune-marker (*M. leprae* PGL-I specific IgM antibodies) with additional cellular immune-markers (IP-10, CCL4 and IL-10) significantly improved distinction between *M. leprae* infected and non-infected individuals^7^. In this setting the BI of most leprosy patients was less than 1 which generally corresponds with the absence of anti-PGL-I antibodies^6,16^. The detection of additional cellular markers increased the sensitivity of the assay for these individuals with 39% compared to the LFA based on antibody detection alone^7^.

In the current study, whole blood samples of leprosy patients, their household contacts (HHC) and endemic controls (EC) were collected in Asia, Africa and South-America to evaluate the diagnostic potential of the previously used LFAs in Bangladesh applying detection of IP-10, CCL4 and PGL-I specific antibodies^7^. Additionally, a new UCP-LFA for detection of C-reactive protein (CRP) was developed and evaluated in these cohorts as CRP, an acute phase protein produced by the liver in response to inflammation, is elevated in LL/BL leprosy patients^17^ and active tuberculosis (TB)^18,19^.

The Asian cohort originated from Guizhou, the province with the second highest leprosy prevalence in China, a country with overall low leprosy endemicity^20^. Patients originated from the Qianxinan and the Guiyang prefecture, with a prevalence of 0.085/10,000 and 0.011/10,000, respectively and mostly MB patients (MB/PB ratio: 8.2). The South American cohort was recruited in the state of Pará, Brazil, a region hyperendemic for leprosy with an annual new case detection rate of 35.34 per 10,000 with an MB to PB patient ratio of 1.932. Active transmission is ongoing in this area, evidenced by the high number of children amongst new cases (6.4% in 2013)^21,22^. The African cohort was collected in Kokosa Woreda (Oromia region) in Ethiopia with a prevalence of 0.32/10,000 and 5.9-fold more MB than PB patients^23^. Thus, this study describes the evaluation of multiple UCP- LFAs for leprosy in low (China), medium (Ethiopia) and hyperendemic (Brazil) settings.

## Results

### Patient cohorts

The extent of humoral and cellular immune responses against *M. leprae* differs within the leprosy spectrum^10^, ranging from predominantly humoral in MB patients to largely cellular immune responses in PB patients. The reported ratio of MB/PB patients as well as the level of endemicity differ between the three regions where the study cohorts were recruited (Table 1).

**Table 1:** Patient characteristics.

	Brazil	China	Ethiopia
LL/BL	30 (31%)	47 (76%)	17 (71%)
BT/TT	41 (42%)	10 (16%)	4 (17%)
Others*	26 (27%)	5 (8%)	3 (12.5%)
age (median (min-max)) years	39.5 (8–78)	35 (13–72)	29 (6–75)
male/ female	52/45	40/18	17/7
Prevalence (per 10,000)	NA	0.085 (Qianxinan) 0.011 (Guiyang)	0.32
New case detection rate (per 100,000)	30.4	NA	NA

Patient characteristics of the Brazilian, Chinese and Ethiopian test cohorts. Patients were stratified by clinical form based on Ridley-Jopling classification. The number of lepromatous leprosy (LL)/borderline lepromatous (BL) and borderline tuberculoid (BT)/tuberculoid (TT) patients are indicated for each group. *Patients that were not classified in one of these two groups (borderline, indeterminate, neural leprosy or not assessed) are referred to as others. The prevalence or new case detection rate is region specific.

LL/BL, on the MB side of the leprosy spectrum, was the major form of leprosy observed in China and Ethiopia (respectively 76% and 71%), whereas in the Brazilian cohort the different forms of leprosy were more equally divided (Table 1). In China and Ethiopia the majority of the patients were male, while in Brazil a more even distribution of males and females was observed (Table 1).

### Diagnostic value of single UCP-LFAs

**Figure 1:**
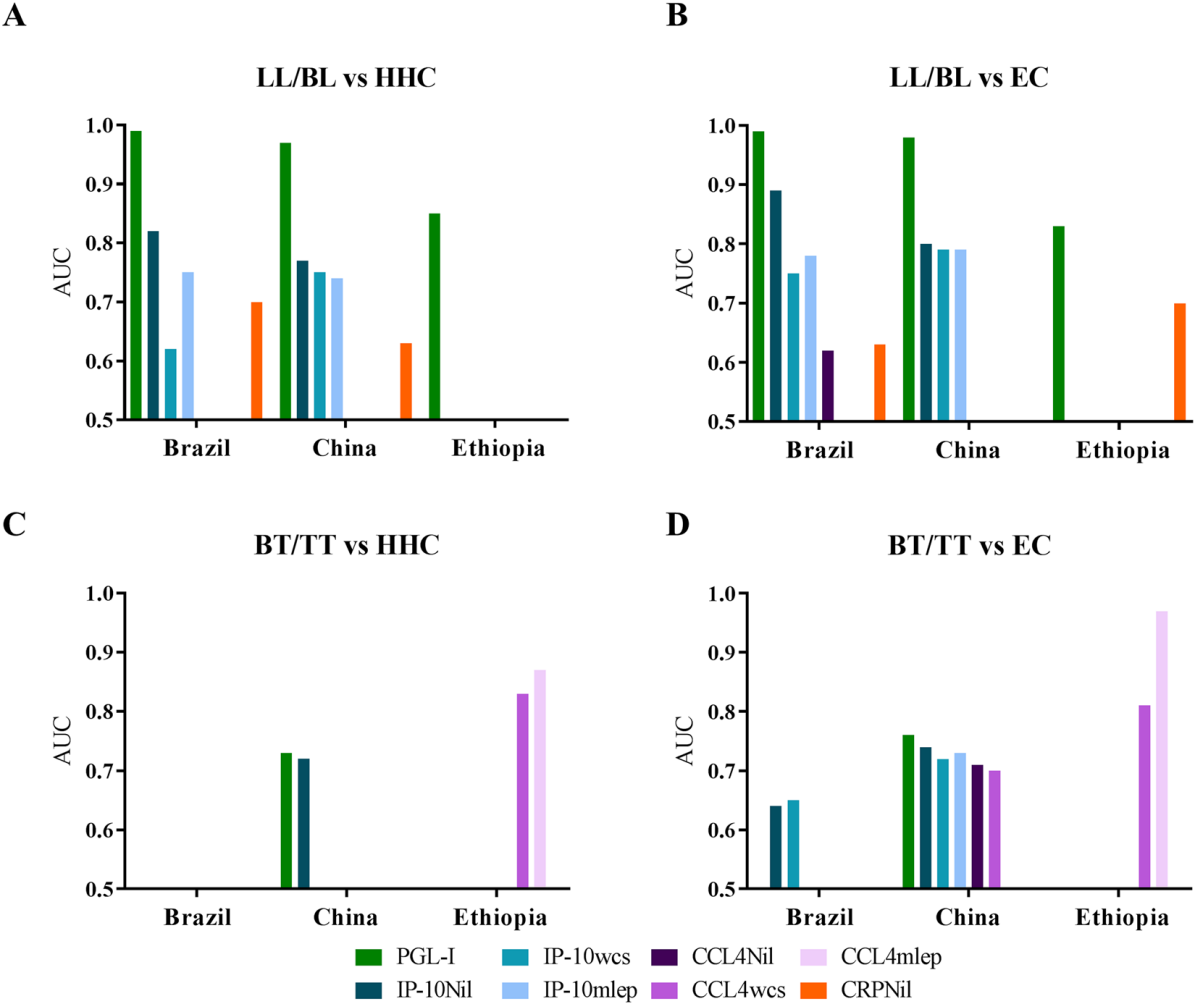
Performance of up-converting phosphor (UCP) lateral flow assays (LFAs). UCP-LFA for detection of anti-PGL-I IgM levels, IP-10, CCL4 and CRP in whole blood (Nil). IP-10 and CCL4 levels were also assessed in whole blood stimulated with *Mycobacterium leprae (M. leprae)* whole cell sonicate (WCS) and two *M. leprae* specific proteins (Mlep). The area under the curve (AUC) was calculated for each individual marker and the significant AUCs are shown per cohort (Brazil, China, Ethiopia). (**A**) Significant AUCs discriminating lepromatous leprosy (LL)/borderline lepromatous (BL) patients from healthy household contacts (HHC). (**B**) Significant AUCs discriminating LL/BL patients from endemic controls (EC). (**C**) Significant AUCs discriminating borderline tuberculoid (BT)/tuberculoid (TT) patients from HHC. (**D**) Significant AUCs discriminating BT/TT patients from EC. China: 47 LL/BL patients, 10 BT/TT patients, 87 HHC and 56 EC. Brazil: 30 LL/BL patients, 41 BT/TT patients, 103 HHC and 237 EC. Ethiopia: 17 LL/BL patients, 4 BT/TT patients, 24 HHC and 25 EC.

Supernatants of WBAs (both *M. leprae* antigen-stimulated, WCS and Mlep, and non-stimulated (Nil)) were used to assess the significance of 4 single UCP-LFAs. The diagnostic potential of each marker (anti-PGL-I IgM, CRP_Nil_, IP-10_Nil_, IP-10_Mlep_, IP-10_WCS_, CCL4_Nil_, CCL4_Mlep_, CCL4 _WCS_) was evaluated based on Ridley-Jopling classifications by assessing the ability of the markers to discriminate LL/BL and BT/TT patients from HHC and EC (Supplementary Table S1). The diagnostic value of each marker was assessed by computing ROC curves, and the associated AUCs (Fig. 1).

In the hyperendemic Brazilian cohort (Supplementary Fig. S1), anti-PGL-I IgM, CRP and IP-10 (Nil, WCS and Mlep) levels differed significantly between LL/BL patients versus HHC and EC as indicated by the significant AUC values (Fig. 1). Similarly, CCL4_Nil_ levels for LL/BL patients and EC in Brazil were significantly different (Fig. 1), while a good distinction between BT/TT patients and EC was provided by IP-10_Nil_ and IP-10_WCS._

In the Chinese cohort (supplementary Fig. S2), anti-PGL-I IgM and IP-10 (Nil, WCS and Mlep) levels significantly differed in LL/BL patients from HHC and EC (Fig. 1). Additionally, CRP levels distinguished LL/BL patients from HHC. BT/TT patients significantly differed from HHC and EC in anti-PGL-I IgM levels and IP-10_Nil_. Moreover, IP-10_WCS_, IP-10_Mlep,_ CCL4_Nil_ and CCL4_WCS_ differentiated BT/TT patients from EC.

In the Ethiopian cohort (Supplementary Fig. S3), anti-PGL-I IgM levels distinguished LL/BL patients from HHC and EC, whereas CRP levels reached significance between these patients and EC. CCL4_WCS_ and CCL4_Mlep_ discriminated BT/TT patients from HHC and EC.

Overall, the data show that IP-10 was the most significant cellular marker to identify both LL/BL and BT/TT leprosy patients in low- and high endemic populations, anti-PGL-I IgM and CRP are relevant for diagnosis of LL/BL patients and CCL4 contributes to the detection of BT/TT patients.

### Identification of multi-biomarker signatures

To identify a biomarker signature specific for leprosy disease in general, we included in this signature besides anti-PGL-I IgM also cellular markers based on the AUCs (Fig. 1).

In the Chinese cohort both IP-10 and CCL4 enabled the distinction between BT/TT patients and EC, with the highest AUC for a single analyte for IP-10_Nil_ and CCL4_Nil_ (Fig. 1). In Brazil, IP-10_Nil_ and IP-10_WCS_ discriminated BT/TT patients from EC, whereas in Ethiopia CCL4_WCS_ and CCL4_Mlep_ showed diagnostic value for these patients. Optimal cut-offs of the selected biomarkers were determined based on the Youden’s index^24^. All individuals positive for both selected cellular markers were designated positive (Supplementary Fig. S4).

**Figure 2:**
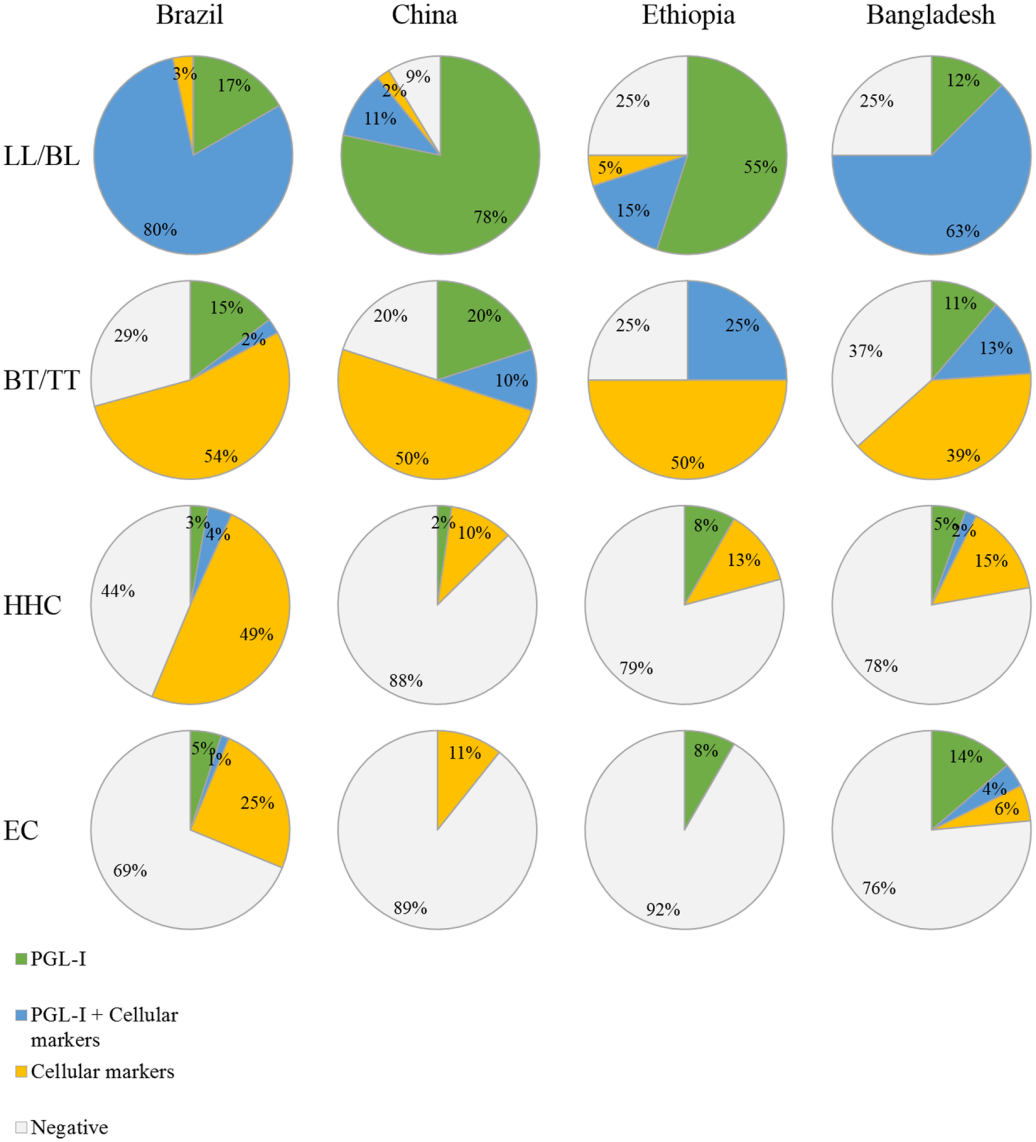
Combination of cellular and humoral markers improves the detection of leprosy patients. Pie charts showing the percentage of individuals with a positive test result for anti-PGL-I IgM (light green), cellular markers (yellow), both anti-PGL-I IgM and cellular markers (blue) or without positive test results (light grey; Supplementary Fig. S4) per test group (lepromatous leprosy/borderline lepromatous (LL/BL), borderline tuberculoid / tuberculoid (BT/TT) patients, healthy household contacts (HHC) and endemic controls (EC)). PGL-I IgM was included to identify LL/BL patients. The threshold for positivity was determined based on the Youden’s index, resulting in a cut-off of >0,205, >0,61 and >1,195 for Brazil, China and Ethiopia respectively for PGL-I IgM. The threshold for positivity was determined as well for two cellular markers that were selected per cohort based on the areas under the curve (AUC) depicted in Fig. 1: CCL4_Nil_ and IP-10_Nil_ (China; cut-off >0,355 and >0,105 respectively), IP-10_Nil_ and IP-10_WCS_ (Brazil; cut-off >0,395 and >0,855 respectively); CCL4_WCS_ and CCL4_Mlep_ (Ethiopia; cut-off <1,03 and <1,13 respectively)_._ China: 47 LL/BL patients, 10 BT/TT patients, 87 HHC and 56 EC. Brazil: 30 LL/BL patients, 41 BT/TT patients, 103 HHC and 237 EC. Ethiopia: 17 LL/BL patients, 4 BT/TT patients, 24 HHC and 25 EC. For comparison data obtained from a previous study performed in Bangladesh, using IP-10_WCS_ and CCL4_WCS_ as cellular markers, was shown (8 LL/BL, 71 BT/TT, 54 HHC and 51 EC).

The use of multi-biomarker signatures consisting of cellular markers and humoral anti-PGL-I IgM seropositivity resulted in four possible outcomes depicted in Fig. 2. With a sensitivity for LL/BL patients of 91%, 97% and 75% in China, Brazil and Ethiopia respectively, the majority of LL/BL patients was identified by the PGL-I UCP-LFA with little added value of the cellular markers identifying 2%, 3% or 5% additional patients respectively in line with the immune responses within the leprosy spectrum.

On the other hand, for BT/TT patients the combination of cellular and humoral markers increased the test sensitivity with 50% to 54%, resulting in an overall sensitivity for BT/TT leprosy of 80% (China), 71% (Brazil) and 75% (Ethiopia). Similar analysis was applied to previously described data from a cohort in Bangladesh^7^, additionally detecting 39% of BT/TT patients resulting in an overall sensitivity of 63%. Importantly, specificity was not relevantly affected by the inclusion of cellular biomarkers in China and Ethiopia and was only moderately decreased in Brazil and Bangladesh. Of the HHCs, 10%, 13%, 15% and 49% were positive for cellular markers in the Chinese, Ethiopian, Bangladeshi and Brazilian cohorts, respectively. These data show that biomarker profiles based on humoral and cellular markers can identify patients at both ends of the leprosy spectrum, irrespective of geographical region.

### Decision tree as a field-tool to assess leprosy risk profiles

**Figure 3:**
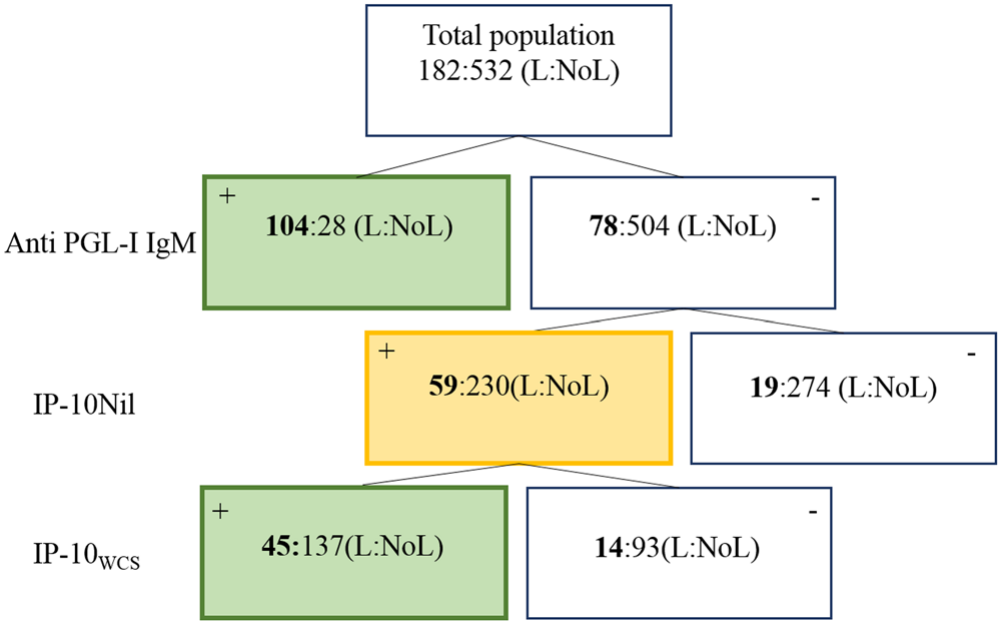
Decision tree as a tool to assess leprosy risk profiles. Decision tree to identify individuals with *M. leprae* infection or at risk of developing leprosy based on the data obtained from all three cohorts. The total population is first stratified by anti-PGL-I IgM levels indicating the total number of individuals positive (left box) and negative (right box) with the number of leprosy patients indicated in bold (L = leprosy; NoL = no leprosy). In the second step, the anti-PGL-I IgM seronegative individuals are stratified by IP-10_Nil_ levels indicating the total number of individuals positive (left box) and negative with the number of leprosy patients (L) indicated in bold. In the third step all individuals positive for IP-10_Nil_ (yellow box) are stratified by IP-10_WCS_ levels indicating the total number of individuals positive (left box) and negative with the number of leprosy patients (L) indicated in bold. The green boxes indicate the individuals that are identified as *M. leprae* infected or at risk of developing leprosy.

For detection of leprosy patients and/or individuals at risk of developing leprosy in field situations, a decision tree based on low-complexity diagnostic tests may facilitate decision making by local health workers. As biomarker based diagnostic tests should be globally applicable, we constructed a decision tree irrespective of cohort applying anti-PGL-I IgM and IP-10 as general biomarkers for leprosy patients. As anti-PGL-I IgM levels measured by UCP-LFA were highly specific for *M. leprae* infection and can be considered a correlate of risk for developing leprosy, this was designated the first step of the decision tree. This step identified 132 individuals (based on the cut-offs described in Fig. 2), among whom 104 leprosy patients (57% of total patients in the 3 cohorts) and 28 individuals without leprosy (5%) (Fig. 3).

In the next step, IP-10_Nil_ levels were used for anti-PGL-I IgM seronegative individuals, which detected an additional 59 patients thereby identifying 89% of all leprosy cases. Of the individuals without clinical leprosy 230 (43%) were IP-10_Nil_ positive. Subsequent assessment of the IP-10 levels in response to *M. leprae* WCS in the IP-10_Nil_ positive individuals increased specificity; while 81% of the patients were identified this step reduced the number of individuals without leprosy that were identified as being at risk to 137 (26%; Supplementary Fig. S5A). Stratifying these data by cohort showed that based on this three-step decision model of the individuals without leprosy 38% in Brazil (n = 131; 46 HHC; 74 EC), 22% (n = 11; 7 HHC; 4 EC) in Ethiopia and 16% (n = 23; 16 HHC; 7 EC) in China are considered at risk of developing leprosy or transmitting *M. leprae* (Supplementary Fig. S5B). Thus, use of a decision tree based on multiple, different types of markers measured in low-complexity assays can therefore guide decision making on who needs (prophylactic) treatment in large-scale screening efforts in field settings.

## Discussion

Diagnostic tests for *M. leprae* infection will provide a useful asset in large scale screening efforts to identify individuals who need prophylactic treatment. In a previous study we demonstrated that the combination of field-friendly UCP-LFAs for leprosy detecting cellular and humoral biomarkers as compared to humoral markers alone, increased the sensitivity for detection of patients in Bangladesh by 32% for MB and 36% for PB cases^7^. The current study investigates the use of similar UCP-LFAs in three other cohorts with different leprosy endemicity in order to evaluate their worldwide applicability as field-friendly, point-of-care tests for leprosy based on combined multiplex detection of biomarker profiles.

In line with previous studies^4,6–8^, anti-PGL-I IgM levels identified the majority of LL/BL patients with high bacillary loads. The detection of cellular markers (IP-10 and CCL4) further improved the sensitivity of BT/TT leprosy patients up to 54%. Elevated levels of CRP in LL/BL (MB) leprosy were in agreement with other reported studies^17^.

Cellular markers thus increased sensitivity of leprosy diagnosis and in particular showed added value for BT/TT patients in the three cohorts compared to assessment of anti-PGL-I IgM seropositivity alone. BT/TT patients at the PB side of the leprosy spectrum worldwide comprise 41% of the leprosy patients, signifying the relevance of cellular markers in leprosy diagnostics^11^. Especially with respect to detection of *M. leprae* infection in the absence of clinical symptoms, in particular relevant for the HHC group, cellular markers also diagnosed *M. leprae* infection more often than anti-PGL-I IgM testing.

The low rate (6%) of anti-PGL-I IgM positivity in the Brazilian HHC contrasted earlier observations from the same region^22^ that indicated a remarkably high 77.6% seropositivity in a comparable student population. This difference is thought to be a consequence of improved assay-specificity of the UCP-LFA used in the current study compared to the earlier applied ELISA^6^: the UCP-LFA format is a virtually background-free reporter technology^25^ thereby detecting *M. leprae* infection with higher specificity.

The rate of positivity for cellular markers in HHC correlated with the level of endemicity ranging from 10% in China (low endemic) to 53% in Brazil (high endemic), which is in agreement with previous findings^26^. In Brazil intense transmission continues in the area of this study as revealed by particularly high rates of leprosy cases amongst children^27^. Moreover, it has been reported that in the majority (19 out of 27) of Brazilian states 50% of the individuals is exposed to high or hyperendemic rates of infection^28^. Thus, the Brazilian EC group tested is therefore not unaffected by *M. leprae*, as these school children are likely to have been in contact with *M. leprae* infected individuals^22^. However, using quantitative signals as measured by UCP-LFA in field-settings, cut-off values are adjustable. This facilitates a stepwise approach that can be accommodated for various diagnostic questions (postexposure prophylaxis, monitoring, classification) each with different sensitivity/specificity requirements.

Factors to be considered for the appropriate applicability of UCP-LFAs based on combined biomarker profiles thus are regional differences in the MB/PB ratio and the level of endemicity. Cellular markers clearly represent valuable diagnostic tools in countries with high percentages of PB patients (i.e. Bangladesh)^11^. The level of cellular markers is more frequently elevated in HHC in regions with high leprosy endemicity as the rate of *M. leprae* infection corresponds with high new case detection^26^. Consequently, HHC resemble BT/TT patients with respect to positivity for certain cellular biomarkers. In this regard, the use of a multi-step decision approach, using initial categorization based on anti-PGL-I IgM seropositivity, followed by additional steps based on a cellular biomarkers can identify more sensitively and specifically those at risk of developing leprosy or transmitting bacteria.

The UCP-LFA format applied in this study facilitates rapid testing based on the presence of selected biomarkers as it is compatible with the use of finger stick blood (FSB). UCP-LFAs could thus serve as a rapid FSB screening test for *M. leprae* infection applicable as triage in large scale screening of HHCs aiming to provide PEP to infected individuals, to reduce transmission by infected but non-symptomatic individuals^12,13^. For leprosy diagnosis, on the other hand, a subsequent test using overnight stimulation with *M. leprae* antigens similar to the Quantiferon TB test^29^ can be applied to increase test specificity for leprosy avoiding unnecessary use of antibiotics.

Longitudinal studies sampling contacts of leprosy patients during yearly follow-up are ongoing. This approach will include intra-individual biomarker comparison of individuals before and at diagnosis of clinical leprosy aimed at identification of biomarker signatures specific for early disease in individuals yet lacking symptoms^7,30^. The biomarker profile investigated in the current study indicates a high level of similarity of the immunological response of BT/TT and HHC based on 4 biomarkers. Longitudinal studies will provide biomarker signatures that can be used as correlates of disease in infected individuals before clinical manifestation of leprosy.

In summary, despite minor differences in biomarker specificity due to levels of leprosy endemicity, this study demonstrates that UCP-LFA rapid tests are well suited for diagnosis of leprosy patients and *M. leprae* infected individuals irrespective of geographical region. Multiplex UCP-LFAs will enable the assessment of biomarker signatures in leprosy endemic areas, which can facilitate guidance of prophylactic treatment within large-scale screening efforts to reduce transmission and disease while limiting administration of antibiotics^13^.

## Materials and Methods

### Study cohorts

Leprosy patients were diagnosed based on histological findings and clinical observations determined by experienced leprologists and a leprosy pathologist as previously described^4,31^. Patients were categorized according to WHO classification (MB/PB) and Ridley-Jopling classification and bacterial indices (BI) were determined. All leprosy patient whole blood was collected at initial diagnosis prior to multidrug therapy (MDT).

#### Brazil

Leprosy patients were diagnosed at URE Marcello Candia, Marituba, Pará. From January 2016 until June 2017 samples were collected from 97 leprosy patients (LL/BL:30, BT/TT:41, other: 26 (BB/Indeterminate (I):6, NA:20)), 103 healthy household contacts (HHC) and 237 endemic controls (EC). The EC group consisted of school children that were screened for signs of leprosy but were not diagnosed with the disease.

#### China

Leprosy patients were diagnosed at Guizhou Provincial Center for Disease Control and Prevention and samples were collected from April 2014 until April 2017 from 62 leprosy patients (LL/BL: 47, BT/TT: 10, other: 5 (BB/I:3, NA:2), 87 HHC and 56 EC. EC were not known to have any prior contact with leprosy or tuberculosis patients.

#### Ethiopia

Patients were collected in Kokosa Woreda (West Arsi zone, Oromia region) in Ethiopia from December 2016 until August 2017. Samples from 24 patients (LL/BL:17, BT/TT:4, neural leprosy: 3), 24 HHC and 25 EC were collected.

### Whole Blood Assays (WBAs)

Upon recruitment venous, heparinized blood (4 ml) was added within 3 hours to vials pre-coated with *M. leprae* whole cell sonicate (designated WCS), ML2478/ML0840 recombinant proteins (designated Mlep)^26^ or without antigen stimulus (designated Nil)^7,30^. After 24 hour incubation at 37 °C, materials were frozen at −20 °C. Before analysis by UCP-LFA^14^ WBA vials were thawed and supernatants removed after centrifugation.

### PGL-I and M. leprae whole cell sonicate (WCS)

The synthetic disaccharide epitope (3,6-di-O-methyl-β-D-glucopyranosyl(1 → 4)2,3-di-O-methylrhamnopyranoside), identical to that found on the *M. leprae* specific PGL-I glycolipid, was coupled to human serum albumin (to produce synthetic PGL-I; designated ND-O-HSA)^32^ and *M. leprae* whole cell sonicate (WCS) generated with support from the NIH/NIAID Leprosy Contract N01-AI-25469 were obtained through the Biodefense and Emerging Infections Research Resources Repository (http://www.beiresources.org/TBVTRMResearchMaterials/tabid/1431/Default.aspx).

### UCP Conjugates

Lateral flow assays were developed and performed using luminescent up-converting reporter particles (UCP) allowing quantitative detection of the targeted biomarker^25,33,34^. Sodium yttrium fluoride upconverting nano materials (85 nm, NaYF_4_:Yb^3+^, Er ^3+^) functionalized with polyacrylic acid were obtained from Intelligent Material Solutions Inc. UCP conjugates were prepared with goat anti-human IgM (I0759, Sigma-Aldrich, St.Louis, Missouri, USA), mouse-anti-IP-10 (BC-50; Diaclone Research, Besancon, France), goat-anti-CCL4 (AF-271-NA; R&D systems, Minneapolis, USA) or mouse-anti-CRP (CRP135; Labned.com, Amstelveen, Netherlands) at a concentration of 125 μg (αIP-10, αCRP) or 25 μg (αCCL4) antibody per mg UCP according to the method described previously^15^.

### LF strips

Lateral flow strips (LF strips) were assembled by mounting 10 mm glass fiber sample/conjugate pad (Ahlstrom 8964), 25 mm laminated nitrocellulose membrane (Sartorius UniSart CN95) and 20 mm absorbent pad (Whatman Cellulose 470) on a plastic backing. Sample pad and absorbent pad each overlap 2 mm with the nitrocellulose, respectively at the beginning and the end. All LF strip components were obtained via Kenosha (Amstelveen, the Netherlands). The nitrocellulose was provided with an assay-specific test (T) line and an upstream Flow Control (FC) line. Ready to use LF strips were stored at ambient temperature in plastic containers with silica dry pad.

For PGL-I strips the nitrocellulose was provided with a test (T) line comprised of synthetic PGL-I (ND-O-HSA, see above) and a flow-control (FC) line comprised of rabbit anti-goat IgG (RαG; G4018, Sigma-Aldrich) at a concentration of 100 and 50 ng per 4 mm width, respectively. UCP reporter conjugate was applied to the sample/conjugate-release pad at a density of 200 ng per 4 mm in a buffer containing 5% (w/v) sucrose, 50 mM HEPES pH 7.5, 135 mM NaCl, 0.5% (w/v) BSA, and 0.25% Tween-20. The pads were dried 1 hour at 37 °C. For IP-10, CCL4 and CRP LF strips the T line comprised mouse-anti-IP-10 mAb (Clone BC-55; Diaclone Research), mouse-anti-CCL4 mAb (MAB271; Clone #24006; R&D systems) or mouse-anti-CRP mAb (Clone C5; Labned.com, Amstelveen, Netherlands) respectively, at a concentration of 200 ng per 4 mm width. The FC line comprised goat-anti-mouse IgG antibody (M8642; Sigma-Aldrich) for IP-10 and CRP LF strips and 100 ng rabbit anti-goat IgG (RαG; G4018, Sigma-Aldrich) for CCL4.

### LFA protocol

10 µl, 1 µl and 0.1 µl WBA supernatant was diluted in high salt lateral flow (HSLF) buffer (100 mM HEPES pH 7.5, 270 mM NaCl, 1% (w/v) BSA, 0.5% (v/v) Tween-20). 50 ul of diluted sample was added to microtiter plate wells and mixed with 250 ng of target-specific UCP conjugate (IP-10, CCL4 and CRP) before target-specific LF strips were placed in the corresponding wells. Immunochromatography was allowed to continue for at least 30 min until dry.

### LF strip analysis

LF strips were scanned locally using portable LF strip readers adapted for measurement of the UCP label (ESEQuant *LFR* reader with 980 nm excitation and 550 nm emission; QIAGEN Lake Constance GmbH, Stockach Germany). LF strips were shipped to the LUMC and re-analysed using a UCP dedicated benchtop reader (UPCON; Labrox, Finland). Results are displayed as the ratio value between Test and Flow-Control signal based on relative fluorescence units (RFUs) measured at the respective lines.

### Ethics

This study was performed according to the Helsinki Declaration as described previously^30^. The national and institutional Research Ethics Committee, IRB or Beijing Tropical Medicine Research Institute, Beijing Friendship Hospital-affiliate of Capital University of Medical Sciences have approved the study protocol (Colorado State University IRB human protocol 15–6340 H; Ethical Appreciation Certificate N° 26765414.0.0000.0018 (Brazil), ethical approval number 3-10/014/2015 (Ethiopia), ethical approval number BJFH-EC/2014-053 (China). Participants were informed about the study-objectives, the samples and their right to refuse to take part or withdraw from the study without consequences for their treatment. Written informed consent was obtained before enrolment. Informed consent was provided by parents/guardians on behalf of all child participants. All patients who were diagnosed with leprosy received free multidrug treatment (MDT) according to national guidelines.

### Data analysis

Graphpad Prism version 7.02 for Windows (GraphPad Software, San Diego CA, USA) was used to perform Mann-Whitney U tests, Kruskal-Wallis with Dunn’s correction for multiple testing, plot receiver operating characteristic (ROC) curves and calculate the area under curve (AUC). The cut-off with the optimal sensitivity and specificity was determined using the Youden’s index^24^.

## Electronic supplementary material

Supplementary Data
