# Supplementary material for: Evaluation of Immunodiagnostic Tests for Leprosy in Brazil, China and Ethiopia
Source: Sci Rep. 2018 Dec 18;8:17920. doi: 10.1038/s41598-018-36323-1 (PMC6298962; doi:10.1038/s41598-018-36323-1)
Supplement: Supplementary file 1 — Supplementary Data [file 41598_2018_36323_MOESM1_ESM.pdf]

EVALUATION OF IMMUNODIAGNOSTIC TESTS FOR LEPROSY IN BRAZIL, CHINA AND ETHIOPIA

Anouk van Hooij[1], Elisa M. Tjon Kon Fat[2], Moises Batista da Silva[3], Raquel Carvalho Bouth[3], Ana Caroline Cunha Messias[3], Angélica Rita Gobbo[3], Tsehaynesh Lema[4], Kidist Bobosha[4], Jinlan Li[5], Xiaoman Weng[6], Claudio G. Salgado[3], John S. Spencer[7], Paul L.A.M. Corstjens[2], and Annemieke Geluk[1]

[1]Dept. of Infectious Diseases and [2]Dept. Cell and Chemical Biology, Leiden University Medical Center, The Netherlands; [3]Laboratório de Dermato-Imunologia, Instituto de Ciências Biológicas, Universidade Federal do Pará, Marituba, Pará, Brazil. [4]Armauer Hansen Research Institute, Addis Ababa, Ethiopia; [5]Guizhou Provincial Center for Disease Control and Prevention, Guiyang, Guizhou, China; [6]Beijing Tropical Medicine Research Institute, Beijing, China; [7] Dept. of Microbiology, Immunology and Pathology, Colorado State University, Fort Collins, USA.

CORRESPONDENCE TO:

Prof. dr. A. Geluk, Dept. of Infectious Diseases, LUMC

PO Box 9600, 2300 RC Leiden, The Netherlands

Tel: +31-71-526-1974; Fax +31-71-526-5267 ; E-mail: a.geluk@lumc.nl

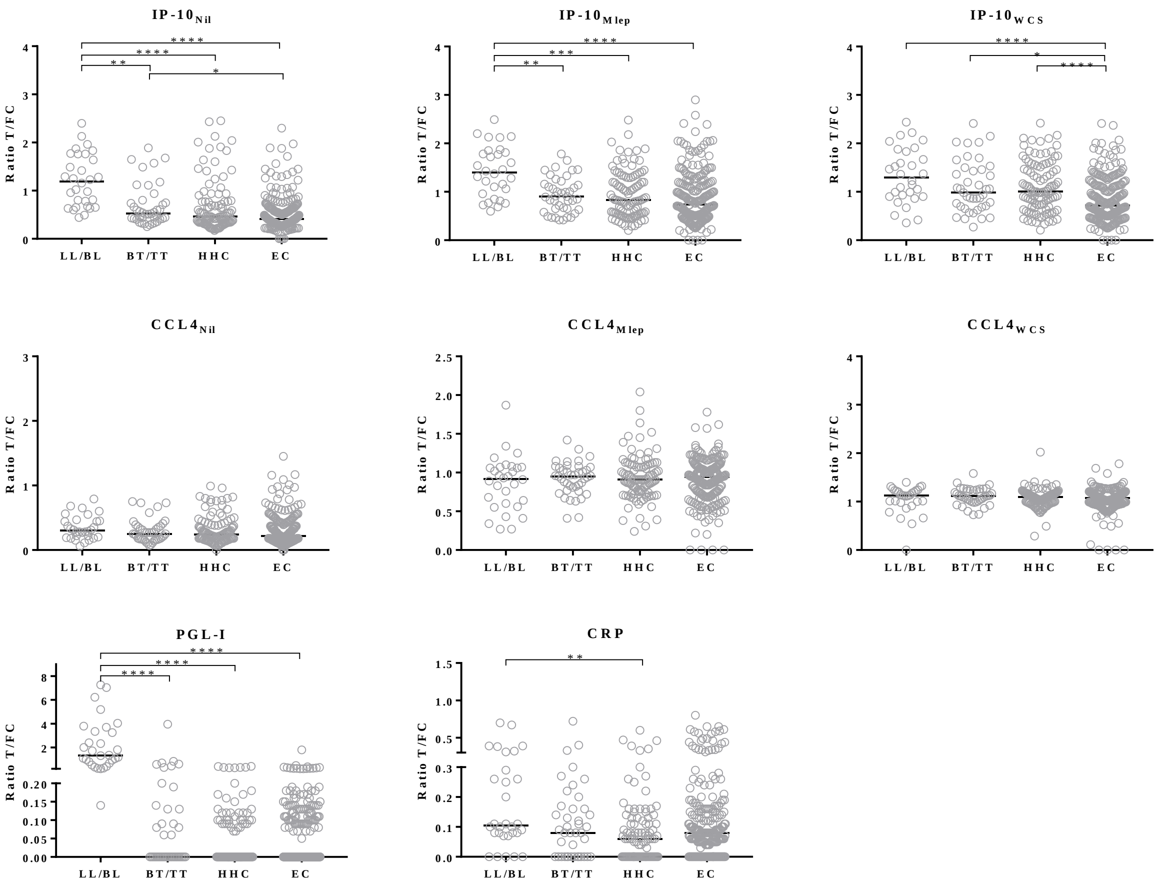

Supplementary Figure S1: Up-converting phosphor (UCP) lateral flow tests (LFAs) performed for the Brazilian cohort. Ratio values of the peak areas of the Test line (T) and flow control (FC) on UCP-LFA strips are shown for IP-10, CCL4, CRP and anti-PGL-I IgM measured in both unstimulated and stimulated whole blood (*M. leprae* whole cell sonicate (WCS) or 2 *M. leprae*-specific recombinant proteins (Mlep)) of lepromatous leprosy/borderline lepromatous patients (LL/BL; n=30), borderline tuberculoid/tuberculoid patients (BT/TT; n=41), healthy household contacts (HHC; n=103) and endemic controls (EC; n=237). P-values were determined by the Kruskal-Wallis test with Dunn's correction for multiple testing. P-values: *$p \leq 0.05$, **$p \leq 0.01$, ***$p \leq 0.001$, ****$p \leq 0.0001$.

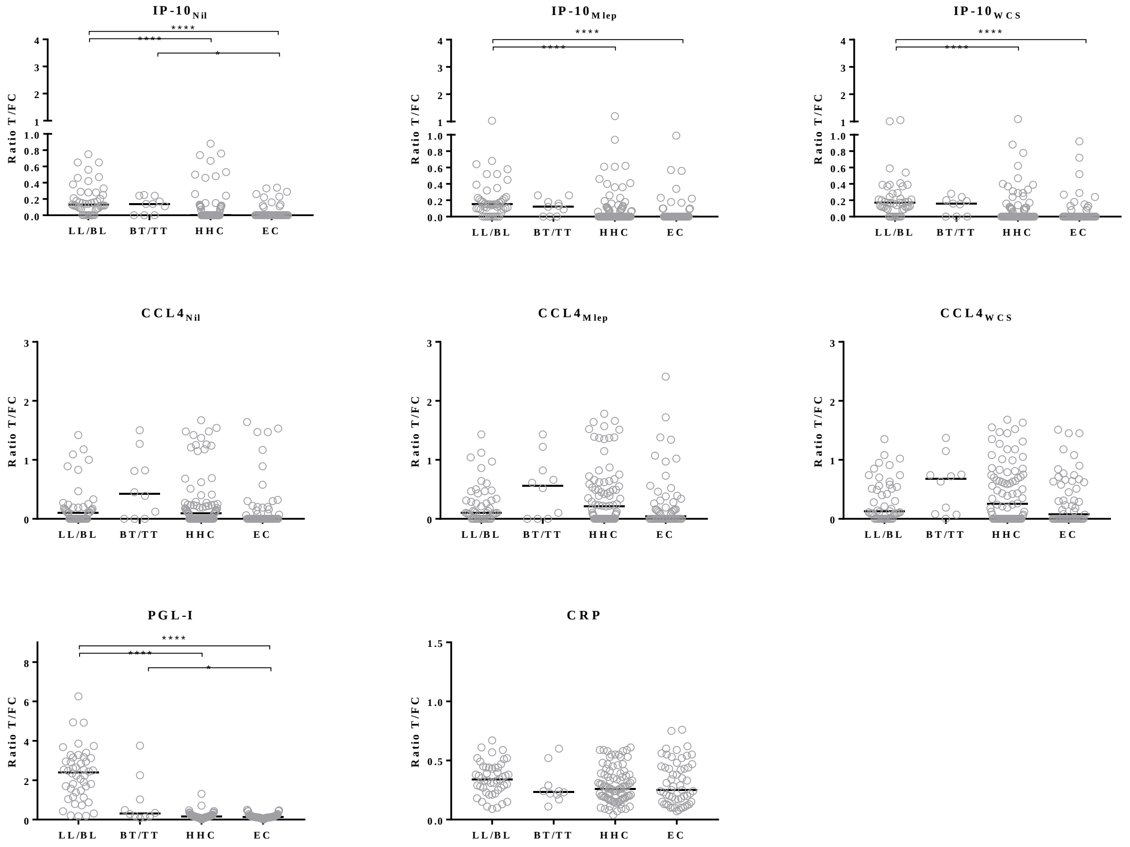

Supplementary Figure S2: Up-converting phosphor (UCP) lateral flow tests (LFAs) performed for the Chinese cohort Ratio values of the peak areas of the Test line (T) and flow control (FC) on UCP-LFA strips are shown for IP-10, CCL4, CRP and anti-PGL-I IgM measured in both unstimulated and stimulated whole blood (*M. leprae* whole cell sonicate (WCS) or 2 *M. leprae*-specific recombinant proteins (Mlep)) of lepromatous leprosy/borderline lepromatous patients (LL/BL; n=47), borderline tuberculoid/tuberculoid patients (BT/TT; n=10), healthy household contacts (HHC; n=87) and endemic controls (EC; n=56). P-values were determined by the Kruskal-Wallis test with Dunn's correction for multiple testing. P-values: *$p \leq 0.05$, **$p \leq 0.01$, ***$p \leq 0.001$, ****$p \leq 0.0001$.

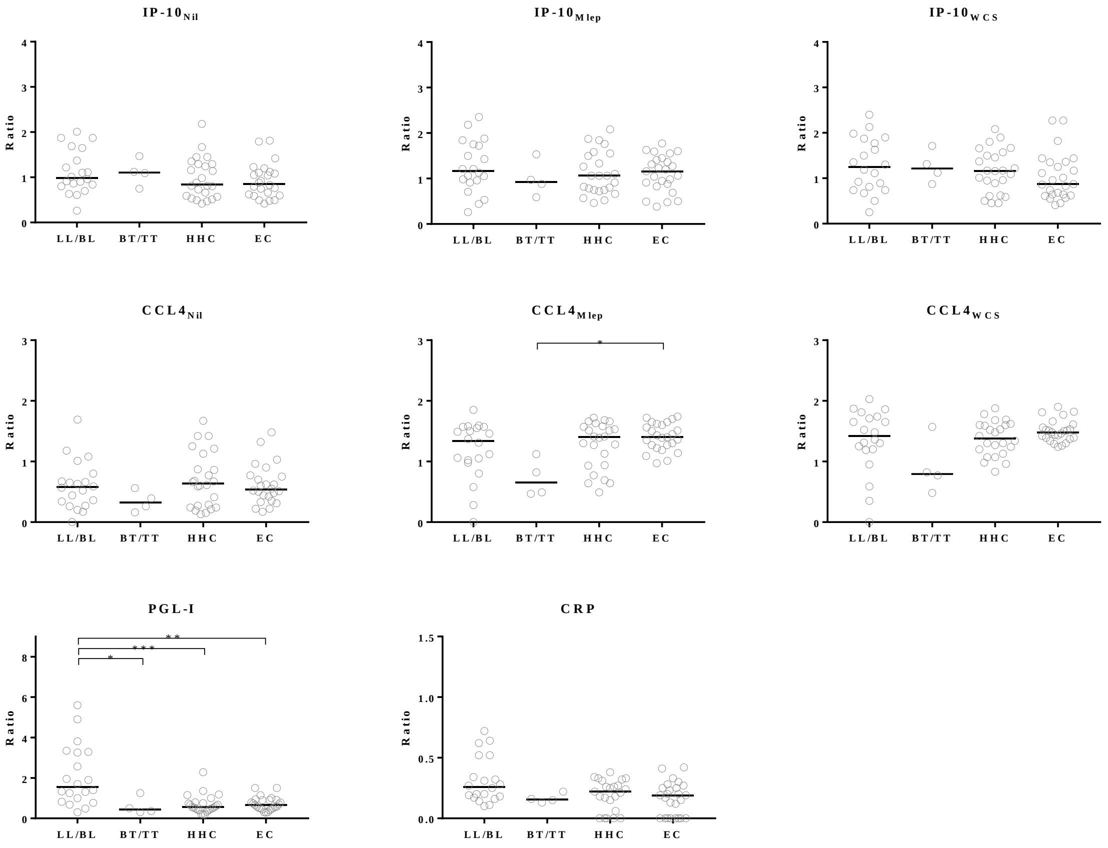

Supplementary Figure S3: Up-converting phosphor (UCP) lateral flow tests (LFAs) performed for the Ethiopian cohort. Ratio values of the peak areas of the Test line (T) and flow control (FC) on UCP-LFA strips are shown for IP-10, CCL4, CRP and anti-PGL-I IgM measured in both unstimulated and stimulated whole blood (*M. leprae* whole cell sonicate (WCS) or 2 *M. leprae*-specific recombinant proteins (Mlep)) of lepromatous leprosy/borderline lepromatous patients (LL/BL; n=17), borderline tuberculoid/tuberculoid patients (BT/TT; n=4), healthy household contacts (HHC; n=24) and endemic controls (EC; n=25). P-values were determined by the Kruskal-Wallis test with Dunn's correction for multiple testing. P-values: *$p \leq 0.05$, **$p \leq 0.01$, ***$p \leq 0.001$, ****$p \leq 0.0001$.

		PGL-I	IP-10_{Nil}	IP-10_{Mlep}	IP-10_{WCS}	$CCL4_{Nil}$	$CCL4_{Mlep}$	$CCL4_{WCS}$	CRP_{Nil}
Brazil	LL/BL vs HHC								
China									
Ethiopia									
Brazil	LL/BL vs EC								
China									
Ethiopia									
Brazil	BT/TT vs HHC								
China									
Ethiopia									
Brazil	BT/TT vs EC								
China									
Ethiopia									
Brazil	LL/BL vs BT/TT								
China									
Ethiopia									
Brazil	HHC vs EC								
China									
Ethiopia									

P-values	
	<0,05-0,01
	<0,01-0,001
	<0,001-0,0001
	<0,0001

Supplementary Table S1: Biomarker potential of anti-PGL-I IgM, IP-10, CCL4 and CRP

Significantly different markers per cohort (Brazil, China, Ethiopia) between two groups as determined by Mann-Whitney U test for lepromatous leprosy (LL)/ borderline lepromatous (BL) patients compared to healthy household contacts (HHC) and endemic controls (EC), borderline tuberculoid (BT)/ tuberculoid (TT) patients compared to HHC and EC, LL/BL compared to BT/TT patients and HHC compared to EC. P-values are indicated by colour coding.

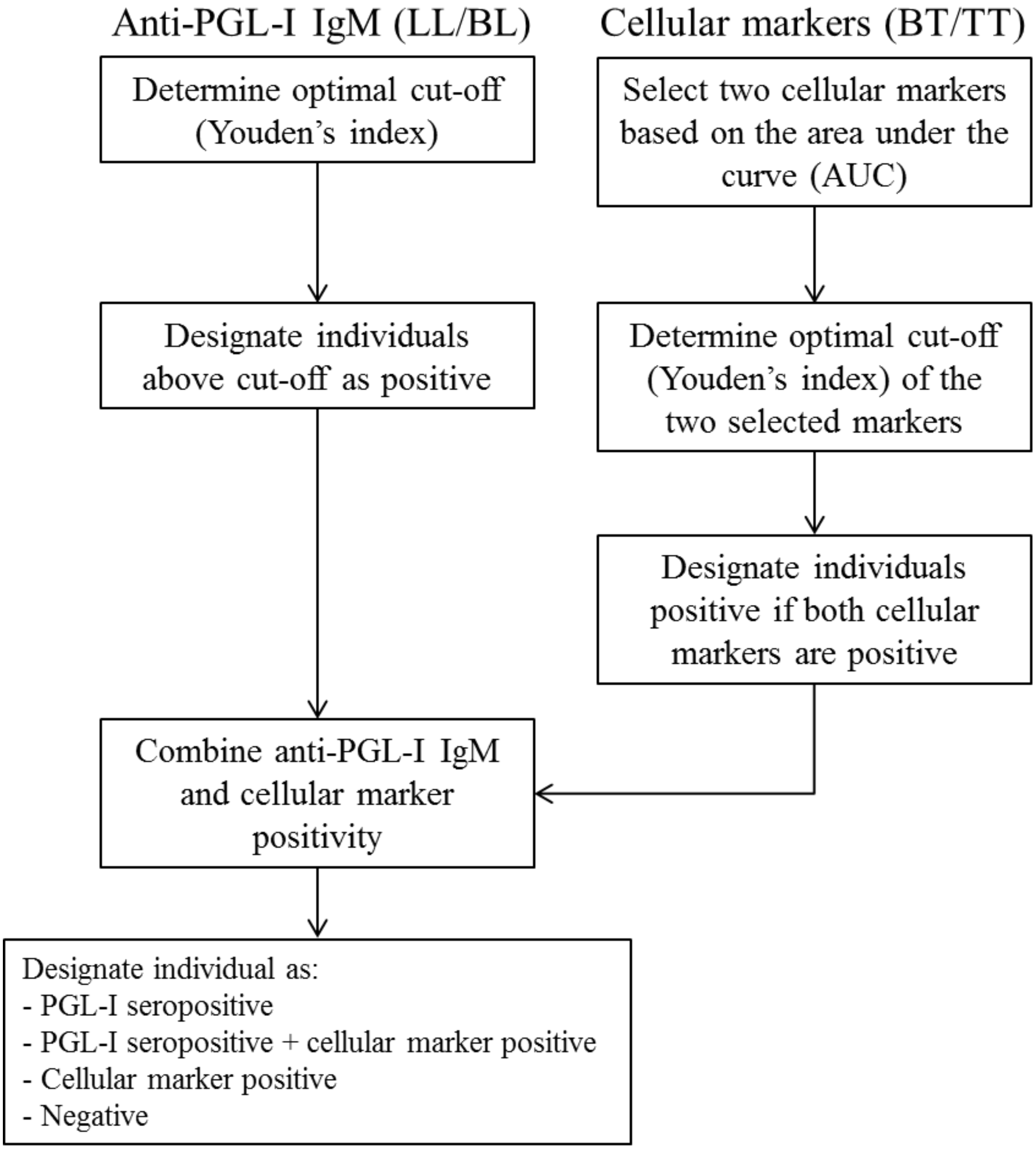

Supplementary Figure S4: Selection procedure of markers. Description of the selection procedures and the combination of cellular and humoral markers (anti-PGL-I IgM) per cohort, resulting in the pie charts in Figure 2.

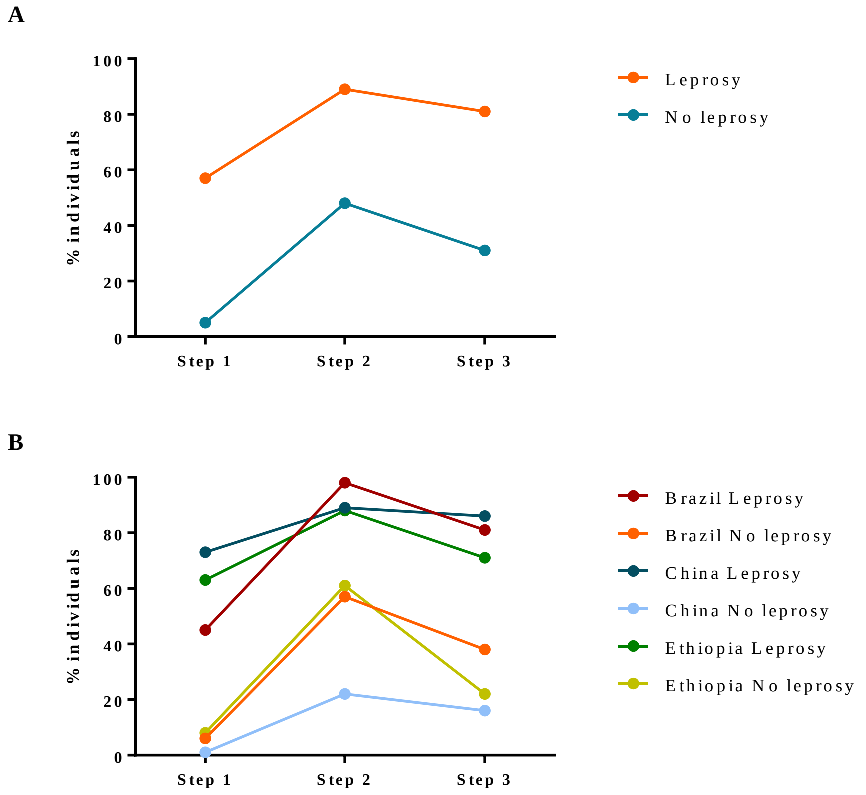

Supplementary Figure S5: Stepwise identification of individuals by a three-step decision tree
Percentage of individuals identified stepwise as positive based on anti-PGL-I IgM (step 1), IP-10_{Nil} (step 2) and IP-10 in response to *M. leprae* whole cell sonicate (step 3) according to the decision tree in Figure 3. (A) Percentage of individuals with (orange) and without (blue) leprosy identified by each step of the decision tree. (B) Percentage of individuals with and without leprosy identified by each step of the decision tree stratified by cohort. Brazil: leprosy (red)/no leprosy (orange), China: leprosy (dark blue)/no leprosy (light blue), Ethiopia: leprosy (green)/no leprosy (light green).
